# A new description of epileptic seizures based on dynamic analysis of a thalamocortical model

**DOI:** 10.1038/s41598-017-13126-4

**Published:** 2017-10-19

**Authors:** H. Sohanian Haghighi, A. H. D. Markazi

**Affiliations:** School of Mechanical Engineering, Iran University of Science and Technology, Tehran, 16844 Iran

## Abstract

Increasing evidence suggests that the brain dynamics can be interpreted from the viewpoint of nonlinear dynamical systems. The aim of this paper is to investigate the behavior of a thalamocortical model from this perspective. The model includes both cortical and sensory inputs that can affect the dynamic nature of the model. Driving response of the model subjected to various harmonic stimulations is considered to identify the effects of stimulus parameters on the cortical output. Detailed numerical studies including phase portraits, Poincare maps and bifurcation diagrams reveal a wide range of complex dynamics including period doubling and chaos in the output. Transition between different states can occur as the stimulation parameters are changed. In addition, the amplitude jump phenomena and hysteresis are shown to be possible as a result of the bending in the frequency response curve. These results suggest that the jump phenomenon due to the brain nonlinear resonance can be responsible for the transitions between ictal and interictal states.

## Introduction

The mathematics of brain dynamics is an interesting, growing and controversial topic. There are two different views about the inherent dynamics of the brain. One view is that the brain function has stochastic dynamics and the irregular patterns of brain signals are due to random activity of neurons. On the contrary, another view states that the electrical signals of the brain are not just a background noise but can be addressed as a quasi deterministic output of the brain activity. The promising fact is that, there are a number of evidences supporting the second hypothesis. However, the brain is a complex network of billions of neurons and a convincing proof of deterministic behavior in such a network is still a challenging problem. At microscopic level, results of the Hodgkin and Huxley^1^ studies show that the behavior of neurons can be better interpreted as a deterministic dynamical system. Based on experimental data, they formulated a model for neuron activity consisting of a set of nonlinear differential equations and received the Nobel Prize for this great achievement. There are also neural population models^2–7^ and neural field models^8^ that are deterministic and developed based on experimental findings and support the view of deterministic dynamics at larger scales. Abrupt switching between different states and the presence of some nonlinear features such as subharmonic entrainment in the brain response to a periodic stimulus are some other signs of deterministic component in the brain activity^9^.

Cortical brain activity can be recorded by electroencephalogram (EEG). In a normal brain state, the recording signal has a complex nature. This kind of behavior in deterministic systems can only be found in chaotic systems. This is the main motivation for further research on finding chaotic attractors in the brain, starting from 1980s^10,11^ and continuing as an interesting field of research in neuroscience^12–20^. While the normal brain activity has an irregular pattern, this is not the case in some abnormal situations like during epileptic seizures in which the quasi periodic waveforms with high amplitude can be observed. Since about one-third of epilepsy patients are drug resistant; it is important to understand the mechanism of transitions between normal and abnormal epileptic states in order to improve prediction algorithms and extend stimulation-based control methods^21–28^. Unfortunately, the cause of such transitions has been poorly understood. In fact, it seems that no single mechanism can describe all dynamic features of various epileptic disorders^29^ and even dynamic explanation of a single type of epileptic seizures is a challenging task. Several models are developed to describe the dynamics of seizure generation and termination^30–41^. These models suggest various dynamic causes for state transitions such as bifurcation, bistability, excitability and intermittency^42^.

Stimulation can induce or terminate a seizure. It can be either a sensory input, e.g., a visual stimulus, or an input to cortex, e.g., an electrical or magnetic stimulus. Moreover, the brain waves usually are described by rhythmic activities in specific frequency bands. For example, alpha rhythm consists of sinusoidal waveforms with frequencies in the range 8–13 Hz and with a peak at around 10 Hz. EEG rhythmic activities have physiological origins^43^ and the observed dominant EEG rhythm depends on the current state of the brain. Different cortical regions also may have different dominant frequencies^44^. So, cortical neural populations can receive nearly periodic inputs from various internal or external sources.

Recent studies reveal that analysis of the neural models subjected to periodic stimulation can explore many nonlinear characteristics of the brain such as nonlinear resonance, chaos, entrainment and nonlinear acceleration^45–49^. In this paper, the frequency response of a thalamocortical model^31^ is investigated to identify some important aspects of the model dynamics and state transitions. Our aim is to find a dynamic explanation for state transitions in the brain, especially for the case of epileptic seizures.

## Materials and Methods

### Neurobiological background

**Figure 1:**
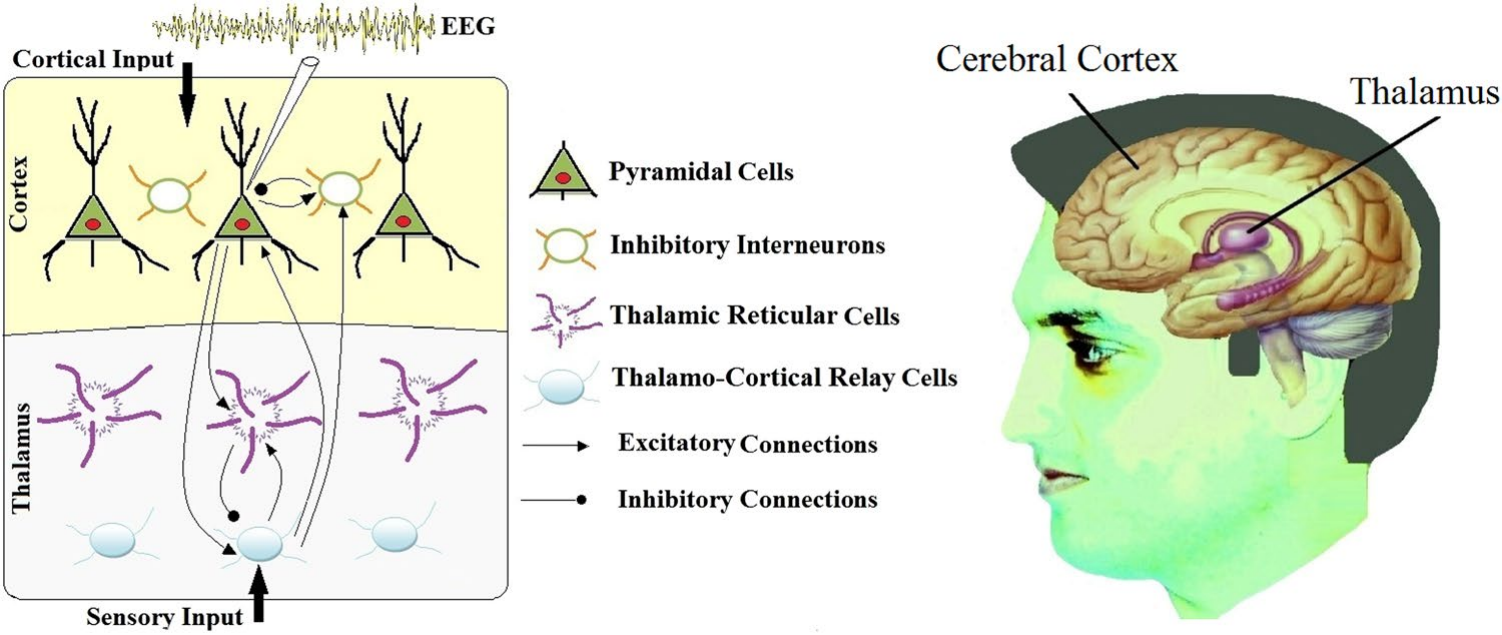
Schematic depiction of the thalamocortical interactions and physiological representation.

EEG data is the main source of information about the dynamics of the brain. The main contributors to the EEG signals include the cerebral cortex and the thalamus. The cerebral cortex contains about half of all nerve cells in the brain and plays an important role in many essential functions of the brain such as sensory and cognitive processes. The majority of neurons in the cortex are pyramidal neurons which are named because of the pyramid like shape of the cell bodies. They are the primary cells that contribute to the EEG signal. Interneurons are the remaining cortical neurons which have various shapes and characteristics. Most of them are inhibitory and can receive both excitatory and inhibitory synapses^50^. Both of these two types of cortical neurons receive sensory inputs from the thalamus. The thalamus is an egg shaped small structure in the central brain region that acts like a relay center and transmits the sensory information to the responsible regions of the cortex. It is composed of various distinct nuclei (neural cell groups) most of which are relay nuclei that relay the sensory information to the cortical neurons. An important and different thalamic nucleus is the thalamic reticular nucleus which has a shell shaped structure and covers the thalamus. The thalamic reticular nucleus modulates the activity of the thalamic relay nuclei but does not send massages to the cortex directly. The neural connections and transmissions are interpreted from biochemical point of view. Inhibitory neurons release the neurotransmitters gamma-aminobutyric acid (GABA). GABA is an inhibitory neurotransmitter because it generates a negative potential and inhibits the excitation of nearby neurons. In contrast, AMPA (α-amino-3-hydroxy-5-methyl-4-isoxazolepropionic acid) is a major excitatory neurotransmitter in the brain and is responsible for most excitatory connections in the thalamocortical system. Figure 1 represents the schema of the thalamocortical system.

### Thalamocortical model

An important model which describes the interactions between the thalamus and the cortex is proposed by Suffczynski and his colleagues^31^. They extend a lumped thalamic model^4^ by adding a cortical model and taking into account the corresponding interactions. The main feature of the model is the ability to describe the spontaneous transition to a seizure like state with characteristics in agreement with the experimental data^51^. The model contains four cell populations: pyramidal (PY) and interneuron (IN) populations for cortex module and thalamocortical relay (TC) and reticular thalamic (RE) populations for thalamus module. Thalamocortical population receives both fast GABA_A_ and slow GABA_B_ inhibition from the reticular thalamic population and send excitatory, AMPA synapses to cortical populations and to reticular thalamic population. Both TC and RE receive excitatory inputs from the pyramidal population. In Cortical area, pyramidal population receives GABA_A_ and GABA_B_ inhibitory synapses from interneuron population and send excitatory AMPA type synapses to it. There are also three external inputs in this model. Sensory input to the TC population, cortical excitatory input to the PY population that represents the inputs from the other pyramidal populations and a constant inhibitory input to RE population which is responsible for the bias input from the other RE populations. The model dynamics is under the influence of the external inputs. Authors, argue that by changing the bias value of the cortical input, the model shows bistability and the fluctuation in the input is responsible for transitions between normal and seizure states.

### Synaptic transmission

The dynamics of the mean membrane potentials described by the following equation^31^:

1 $$\begin{array}{rcl}{C}_{m}\frac{d{V}^{(i)}}{dt} & = & -\sum {I}_{syn}^{(i)}-{g}_{leak}({V}^{(i)}-{V}_{leak}^{(i)})\\ \quad \,\quad \,\,\,i & = & \{PY,IN,TC,RE\},\\ \quad \,\,\,\,syn & = & \{AMPA,GAB{A}_{A},GAB{A}_{B}\}\\ \,\,\quad \,\,{I}_{syn}^{(i)} & = & {g}_{syn}({V}^{(i)}-{V}_{syn})\end{array}$$

Here, V^(i)^ is the membrane potential corresponding to the population i C_m_, is the membrane capacitance, g_leak_ is the leak current conductance, g_syn_ is the synaptic current conductance and V_leak_ and V_syn_ are the reversal potential of the leak current and the synaptic current, respectively. Synaptic conductance is expressed as a convolution of synaptic impulse response (h_syn_(t)) and a firing density (F(t)) which is responsible for the incoming action potential:

2 $$\begin{array}{l}{g}_{syn}(t)={\int }_{-\infty }^{t}{h}_{syn}(t-\tau )F(\tau )d\tau \\ {h}_{syn}(t)={A}_{syn}[{e}^{-{a}_{1syn}t}-{e}^{-{a}_{2syn}t}],{a}_{2syn} > {a}_{1syn}\end{array}$$

where A_syn_, a_1syn_ and a_2syn_ are amplitude, rise and delay times, respectively. Based on experimental results, a nonlinear relation between the amplitude of the GABA_B_ postsynaptic current and firing density of the RE and IN populations is considered. So, the GABA_B_ current is expressed by:

3 $${I}_{GAB{A}_{B}}={g}_{GAB{A}_{B}}(t)B(F)(V-{V}_{GAB{A}_{B}})$$

where F is the mean firing rate which can be obtained from the mean membrane potential using a sigmoid function:

4 $$F(V)=\frac{{G}_{F}}{1+{e}^{{\nu }_{F}(V-{\theta }_{F})}}$$

where ${G}_{F},\,{{\rm{\theta }}}_{F}$ and ${{\rm{\nu }}}_{F}$ are the maximum firing rate, threshold and slope parameter, respectively. The nonlinear activation function is also a sigmoidal function:

5 $$B(F)=\frac{1}{1+{e}^{{\nu }_{B}(F-{\theta }_{B})}}$$

where ${{\rm{\theta }}}_{B}\,$and *v*_B_ are the activation threshold and slope parameter, respectively. In order to model the low threshold spikes in thalamic populations, two new sigmoidal functions n_inf_ (V) and m_inf_ (V) with their own thresholds and slope parameters should be included. The pulse densities for burst firing F_B_ of the thalamic cells are:

6 $${F}_{B}(V)={G}_{B}{m}_{\inf }(V){\int }_{-\infty }^{t}{h}_{n}(t-\tau ){n}_{\inf }(V)d\tau $$

**Figure 2:**
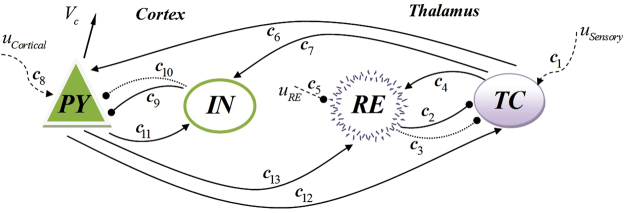
Schematic of the thalamocortical model. The figure shows couplings between the cortical and thalamic modules. Inputs are shown by dashed lines; arrows indicate excitatory connections; circle ends show inhibitory connections and dotted lines indicate slow inhibition.

In this equation, G_B_ is the burst frequency of fast action potentials and h_n_(t) is the the delay function:

7 $${h}_{n}(t)=N[{e}^{-{n}_{1}t}-{e}^{-{n}_{2}t}],{n}_{2} > {n}_{1},N=\frac{{n}_{1}{n}_{2}}{{n}_{2}-{n}_{1}}$$

where n_1_ and n_2_ are decay and rise time, respectively. The model also includes the couplings constants c_1_–c_13_ which are representative of the mean number of synaptic contacts between different populations and time delays between the cortical and thalamic modules. It has three external inputs including sensory input to TC population, cortical input from other parts of the brain to PY population and reticular input to RE population. The model output, (V_c_), is the membrane potential corresponding to PY population. Figure 2 shows schematic of the model and its connections.

### Numerical simulation

Due to the strong nonlinearity and high order of the model, analytical study of the model is very difficult if not impossible. So, in order to explore the model dynamics from input-output point of view, we focused on the effects of the model inputs on its output. For each of both cortical and sensory inputs, a biased sinusoidal waveform signal is considered instead of the biased white noise which was considered in the original model^31^. For RE input, however, a constant signal is used as in the original model. So, the model inputs in the current study are completely deterministic and can be expressed by:

8 $${u}_{sensory}={\phi }_{bs}+{\phi }_{as}\,\sin (2\pi {f}_{s}t)$$

9 $${u}_{cortical}={\phi }_{bc}+{\phi }_{ac}\,\sin (2\pi {f}_{c}t)$$

10 $${u}_{{\rm{R}}{\rm{E}}}={\phi }_{RE}$$

where ${{\rm{\phi }}}_{{\rm{bs}}},{{\rm{\phi }}}_{{\rm{as}}}\,\,$and f_s_ are the bias, amplitude and frequency of the sensory input, and ${{\rm{\phi }}}_{{\rm{bc}}}$,${{\rm{\phi }}}_{{\rm{ac}}}$ and f_c_ are the bias, amplitude and frequency of the cortical input. Also, ${{\rm{\phi }}}_{{\rm{RE}}}\,\,$is the constant reticular input. To investigate the influence of these parameters on the model dynamics, bifurcation analysis is carried out by varying each parameter independently while the others are held constant. Results of the bifurcation analysis for the amplitude parameters, however, are not very informative because they are obtained at specific frequencies. Instead, the simulation results for two different amplitudes in each applicable case are considered here. The final system states of the previously iterated value of the bifurcation parameter are chosen as the initial conditions for the system integration in the next step with the new value of the parameter. The model equations are solved numerically by using Runge-Kutta-Fehlberg method with a sufficiently small relative tolerance. Model parameters remain unchanged with respect to the original model^31^.

### Data availability

No datasets were generated or analyzed during the current study.

## Results

**Figure 3:**
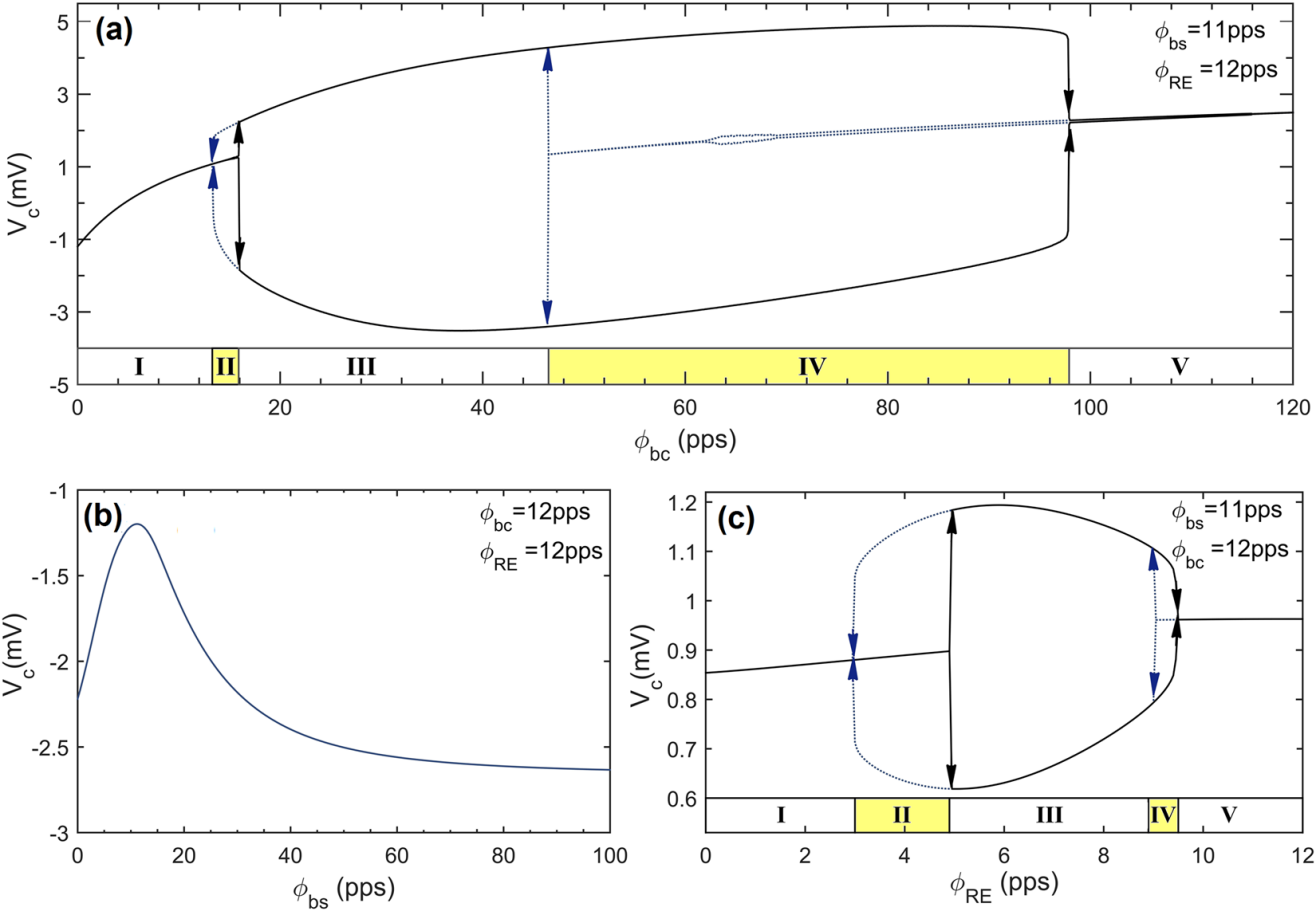
Maximum and minimum output values versus the bias components of the inputs in the absence of sinusoidal components. **(a)** Extrema of output values as a function of the bias component of the cortical input. **(b)** Output values versus the bias component of the sensory input. **(c)** Extrema of output values versus the constant reticular input. Note that the directions of parameter changes are important in diagrams **(a)** and **(c)** because the model output depends on the initial conditions in bistable regions. Simulations were performed in the absence of sinusoidal components.

It was shown that the thalamocortical model dynamics depend on the strength of cortical input. In the absence of noise, as the bias value of cortical input changes, the network dynamics depicts a transition between a stable fixed point and a limit cycle attractor^31^. Systematic bifurcation analysis in a wider range of variables explores more details and additional bifurcations not reported before. Scanning the parameters ${{\rm{\phi }}}_{\mathrm{bc}}\,$and ${{\rm{\phi }}}_{{\rm{RE}}}\,\,$separately in steps of 0.1pps, five main dynamic regimes can be identified for each parameter scan as shown in Fig. 3a and Fig. 3c. In regions I and V there is a stable fixed point and system stays in its equilibrium point. Regions II and IV are bistable regions in which depending on the initial conditions, the system can either go to the equilibrium point or to the high amplitude oscillatory state corresponding to the limit cycle attractor. In region III, oscillating behavior of the membrane potential is the only possible option as a result of the fixed point being disappeared and the limit cycle attractor being kept. As can be seen, some of the regions in Fig. 3a contain more detailed sub-regions, a matter which is out of the scope of this study. Figure 3b reveals that the bias component of the sensory input ($\,{\phi }_{bs}$) does not affect the nature of the system dynamics, but may change the equilibrium.

**Figure 4:**
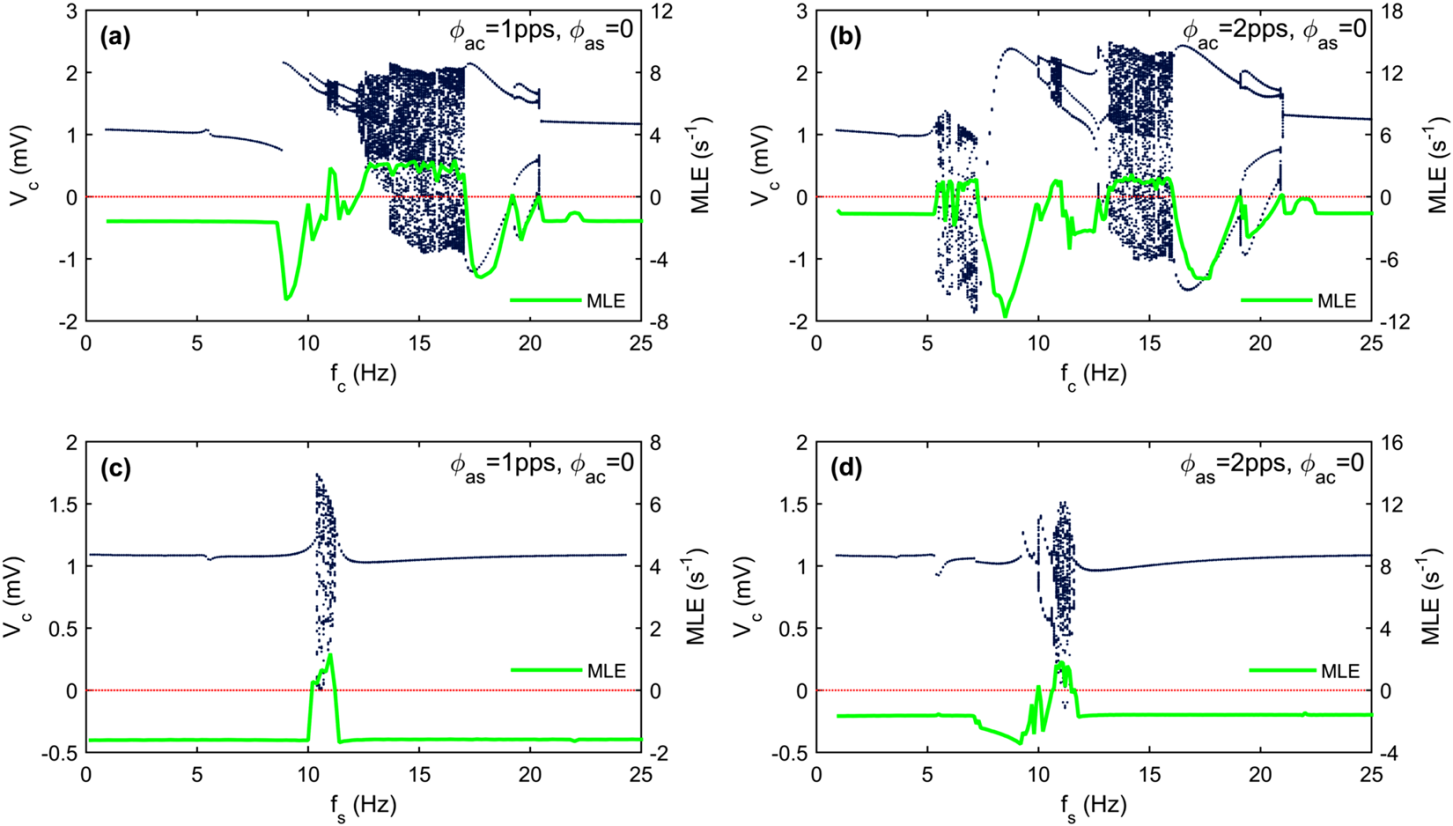
Bifurcation diagrams (dark blue dots, left axis) and maximal Lyapunov exponent (solid green line, right axis) are obtained by varying the frequency of each input while the other inputs are held constant and $\,{\phi }_{bc}=13.5\,{\rm{pps}},\,{\phi }_{bs}=11\,{\rm{pps}}$ and $\,{\phi }_{RE}=12\,pps$. According to Fig. 3a, the selected set of bias inputs indicates a bistable region (region II). Note that, the maximal Lyapunov exponent (MLE) becomes positive in regions within which chaotic behavior occurs.

Next, bifurcation analysis of the thalamocortical model is performed with a focus on the effects of the sensory and cortical input frequencies. The obtained results are useful for finding the regions with different dynamic activity types and getting more insight into the model behavior under periodic inputs. Figure 4 shows the results of bifurcation analysis for four independent cases and with a step increment of 0.1 Hz in each case. Figure 4a reveals the qualitative behavior of the model output versus the cortical input frequency. At low frequencies, a single period is observed. A jump is observed near the frequency of ${{\rm{f}}}_{{\rm{c}}}\approx 9{\rm{Hz}}$ which is the dominant frequency during the seizure like activity in the original model^31^. As the bifurcation parameter is increased further, the system undergoes period doubling bifurcations and also chaotic behavior. The system then undergoes reverse bifurcations, followed by period doubling bifurcation at each branch and then returns to periodicity. To investigate the effects of cortical input amplitude, the bifurcation analysis is repeated for the case ${{\rm{\phi }}}_{{\rm{ac}}}=2{\rm{pps}}$. As it is shown in Fig. 4b an additional chaotic region appears in the bifurcation diagram which starts near the frequency of ${{\rm{f}}}_{{\rm{c}}}\approx 5.5{\rm{Hz}}$ and ends at frequency of ${f}_{{\rm{c}}}\approx 7.5{\rm{Hz}}$. Results of bifurcation analysis by varying the frequency of sensory input are presented in Fig. 4c and Fig. 4d for two different amplitudes of stimulation. Chaotic region appears and disappears suddenly for the case ${{\rm{\phi }}}_{{\rm{as}}}=1{\rm{pps}}$ while for the case ${{\rm{\phi }}}_{{\rm{as}}}=2{\rm{pps}}$ a period doubling bifurcation leads to a chaotic behavior. In each diagram, the maximal Lyapunov exponents are also plotted to confirm the existence of chaotic regimes.

**Figure 5:**
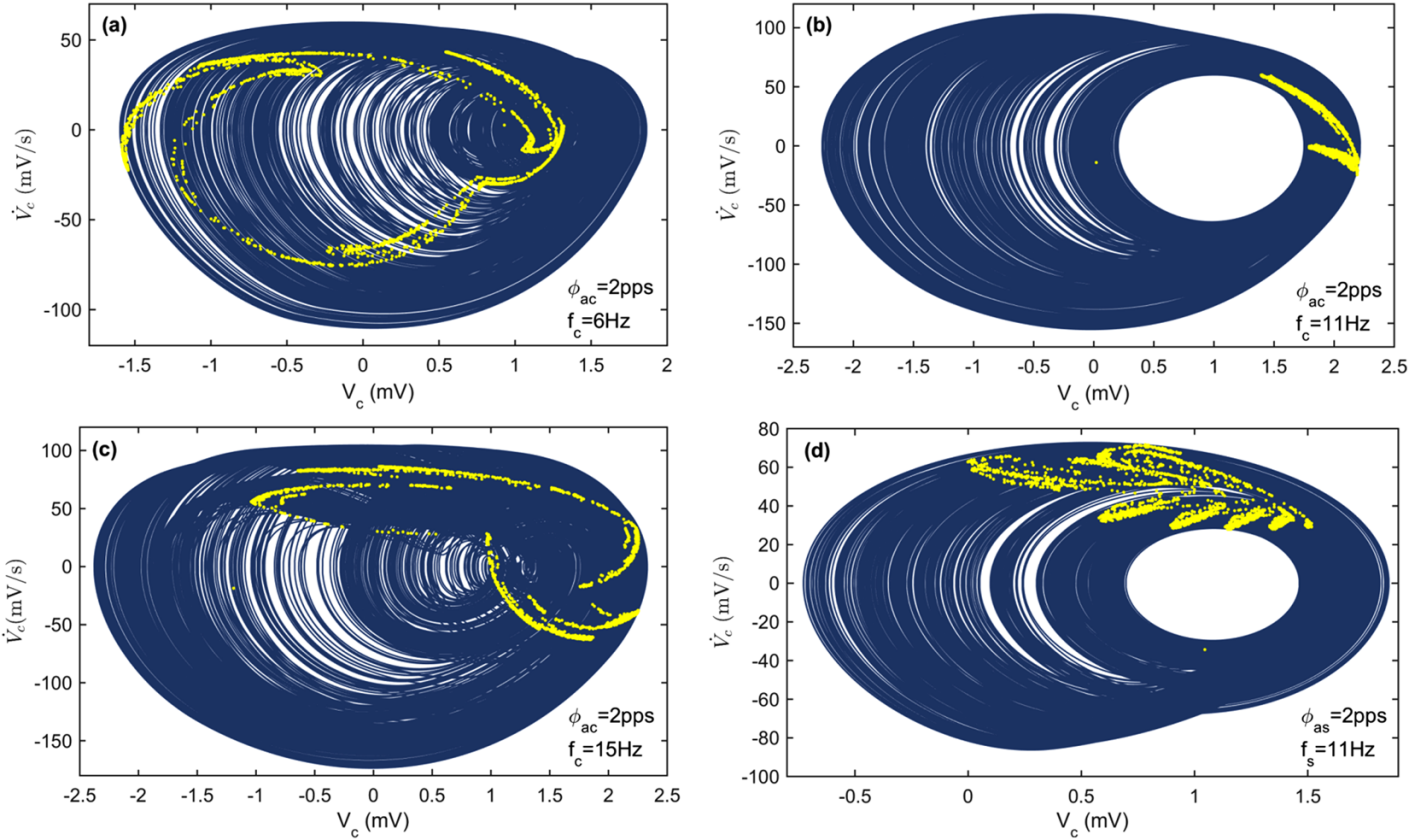
Phase portrait (solid blue) and the corresponding Poincare map (yellow dots) for four different cases. Constant parameters are as in Fig. 4. The time derivative of the output is shown by a dot over the cortical output symbol. Complicated shapes of the Poincare maps confirm the existence of strange attractors.

**Figure 6:**
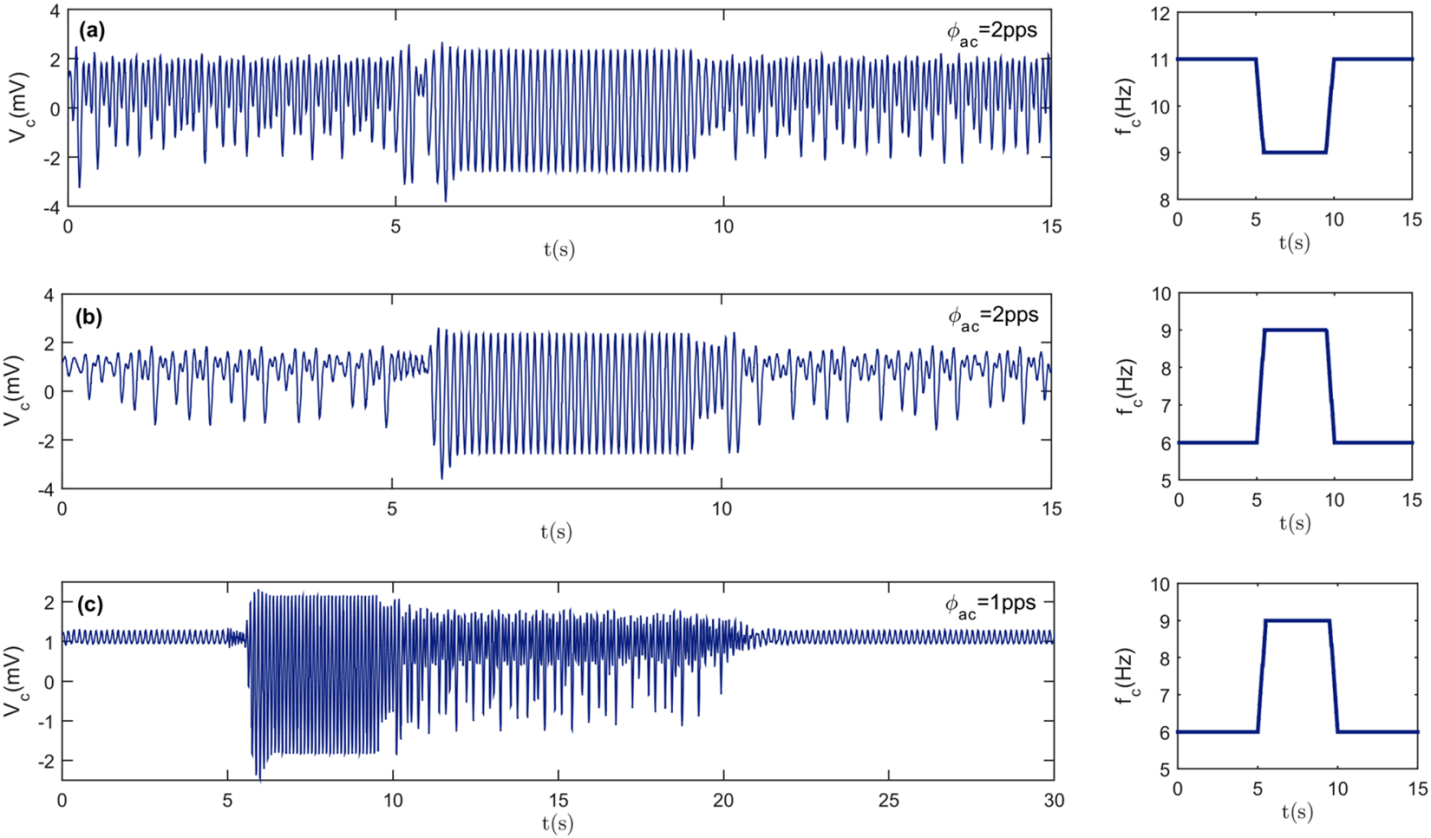
State transitions by changing the cortical input frequency. Constant parameters are as in Fig. 4, ${{\rm{\phi }}}_{{\rm{as}}}=0$ and patterns of cortical input frequencies, f_c_, are presented on the right-hand side diagrams.

The phase portraits with chaotic attractors corresponding to the main chaotic regions in Fig. 4 are separately given in Fig. 5. The results show that different chaotic regions have completely different attractors which indicate the key role of the input parameters. In addition, the bifurcation analysis illustrates that the change in stimulus parameters especially the input frequency may lead to brain state transitions. Transitions between chaotic and periodic regions and also transitions between low and high amplitude periodic regimes are of special interest, because these transitions can be useful in explaining the characteristic features of some neurological disorders such as epilepsy. It can be seen in Fig. 6 that when the cortical input frequency is changed, state transition occurs. By changing *f*_*c*_ from 11 Hz to 9 Hz or from 6 Hz to 9 Hz, the chaotic behavior of cortical output is rapidly replaced by periodic large amplitude oscillation and then the output returns to the chaotic states as f_c_ comes back to its initial value. Figure 6c reveals a transition from small amplitude oscillation to a large amplitude periodic output. After the input frequency returns to the initial value, a transient chaotic response is observed before the system returns to a small amplitude oscillation.

**Figure 7:**
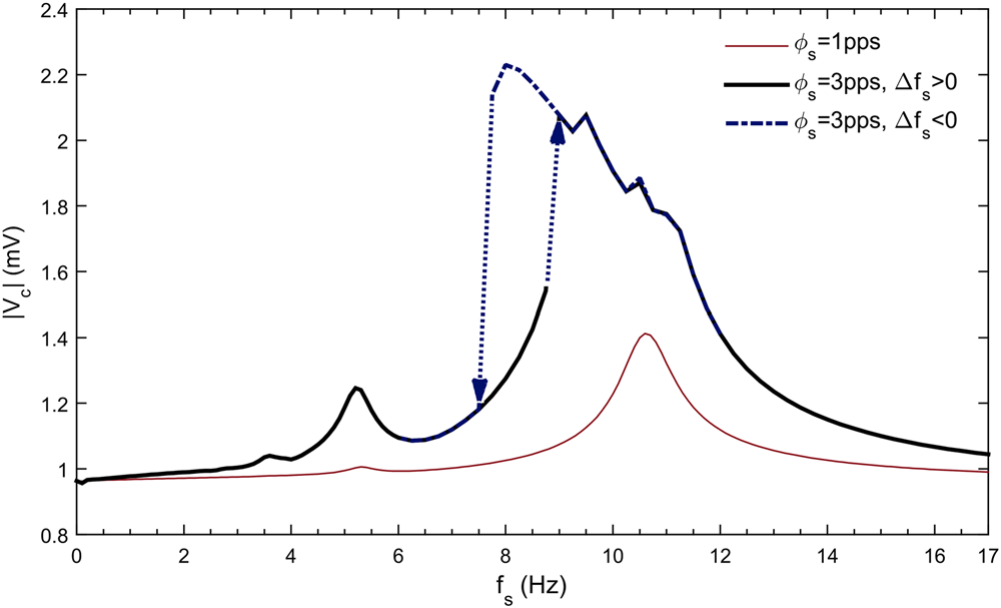
The frequency response of the thalamocortical model subjected to sensory input. Constant parameters are:${{\rm{\phi }}}_{{\rm{bc}}}=12{\rm{pps}},{{\rm{\phi }}}_{{\rm{bs}}}=11\mathrm{pps}\,$,${{\rm{\phi }}}_{{\rm{RE}}}=12{\rm{pps}}$ and ${{\rm{\phi }}}_{{\rm{ac}}}=0\,\mathrm{pps}$. Note that ${\rm{\Delta }}{f}_{s} > 0$ indicates forward sweep frequency response while ${\rm{\Delta }}{f}_{s} < 0$ represents a backward sweep.

According to Fig. 3b in the absence of harmonic excitation, if ${{\rm{\phi }}}_{{\rm{bc}}}=12{\rm{pps}}$ and ${{\rm{\phi }}}_{{\rm{RE}}}=12{\rm{pps}}$, then the system remains in its equilibrium state. In order to investigate the response of the system to a periodic stimulus in this condition, the frequency response of the thalamocortical model subjected to a sensory thalamic stimulation is presented in Fig. 7. For small amplitude stimulus, the model behaves like a linear resonator, while the large stimulus amplitude leads to nonlinear behavior as revealed in the frequency response. For the latter case, subharmonic resonances, bending of frequency response curve, jump phenomena, multi-stability and hysteresis are all observed in the frequency response.

## Discussion

In this paper, we studied the dynamics of a thalamocortical model subjected to external inputs. Biased periodic inputs were considered for cortical and sensory inputs and the effects of bias, frequency and amplitude of stimulation on the behavior of the model are numerically investigated. Several important results were observed. First, bifurcation results show that in the absence of time varying parts of the inputs bistability can occur in two distinct ranges of values for both constant cortical input and constant reticular input. The DC component of sensory input, however, does not cause a bistable regime. Second, stimulation parameters including frequency and amplitude of the stimulus have significant effects on the dynamic behavior of the thalamocortical model output. As an example, it was shown that a change in frequency of cortical input can lead to transitions between chaotic and periodic behavior in the output. Third, even for a set of parameters that are far from the bistable regions for static inputs, bistability can occur in the frequency response of the model. The intensity of stimulation has significant effects on the frequency response of the thalamocortical model. In the following, the importance of these results will be discussed from different viewpoints.

### Complex dynamics of the brain

While existence of chaos in the brain’s EEG is still a controversial topic, chaotic type dynamics have been reported in many neural models such as the Hodgkin- Huxley^52^, the FitzHugh–Nagumo^53^, the Hindmarsh-Rose^54^, the Jansen-Rit^55^ and etc. The results of the present study confirm that the thalamocortical system may have different dynamic regimes including chaos. Changes in the input parameters are required for transition between various regimes which can be provided by a sensory input (e.g. auditory^56^ or visual^57^) or cortical stimulation^58^.

### The brain as a resonator

Frequency response of the thalamocortical model reveals that the brain response to a repetitive stimulation is frequency dependent. This fact have been already reported in some experimental findings^59^. Evidences for resonance in the brain are provided by researchers using various brain stimulation techniques including sensory stimulation^55,60,61^, transcranial magnetic stimulation^62^ and transcranial alternating current stimulation^63^. A recent study concludes that a thalamocortical resonant frequency of about 10 Hz can be observed in both humans and animals^64^. This frequency is the peak frequency in alpha band frequency, which is dominant during relaxation with closed eyes or in the dark. It is not, however, the only one that may reveal resonance in the brain. For example, when the eyes are open, the alpha rhythm attenuates and higher frequencies in the beta range appear. Oscillatory activity of resting human brain may show a peak frequency at about 17 Hz in the beta frequency range^44^. Experimental results of photic stimulation also suggest a resonant frequency about the beta peak frequency (~15 Hz)^65–67^ or around the alpha peak frequency (~10 Hz)^60,68–71^. Such different reports seem to be a consequence of different experimental conditions including eyes condition, stimulation intensity and the ambient light^70,72^, that may lead to the excitation of different dynamic modes in the brain. However, the thalamocortical model used in this paper is based on many simplifications of real brain networks and does not consider all rhythms of the brain. Results of this study indicated that for small amplitude stimulation, the brain responds as a linear resonant system and the response would show a peak at a certain frequency. Moreover, the amplitude of stimulation can change the natural frequency and cause a nonlinear resonance in the neural system as it was reported by some other researchers^46^. Subharmonic resonances also observed in Fig. 7, which is consistent with obtained results in some experiments^68,70,73^. These results confirm the role of stimulation intensity in the brain physiological responses during rhythmic excitation^71,74^. So, the results of current study suggest that the brain responds to a stimulus like a resonator. As the stimulus intensity increases, this resonator behaves more nonlinearly. Bistability and hysteresis are expected to be found in the frequency response of the brain, which can be observed experimentally by sweeping the stimulation frequency forward and backward. Stimulation could be sensory or cortical, for which the intensity has to be chosen wisely to guarantee a safe and effective experiment.

### Dynamic description of epileptic seizures

**Figure 8:**
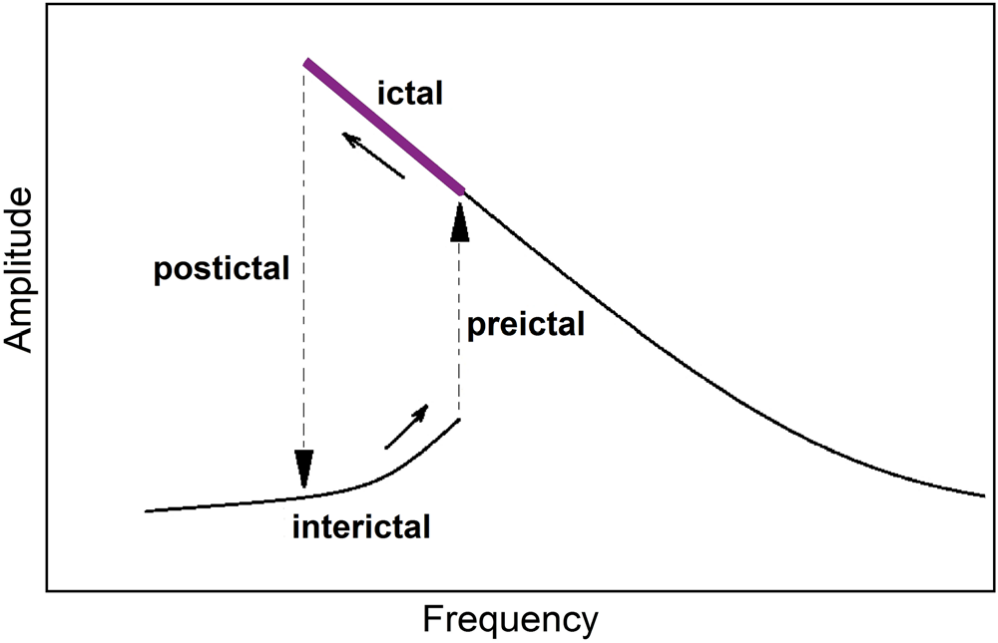
Interpretation of nonlinear resonance as a dynamic mechanism of epileptic seizures. Based on this model, transitions between ictal and interictal states occur because of jump phenomenon due to the brain nonlinear resonance.

The bistability and jump phenomena in the frequency response of the thalamocortical model can be a key in understanding the dynamic mechanism of epileptic seizures. In this view, an epileptic brain responds to a periodic stimulus like a nonlinear resonator, e.g., a softening type duffing oscillator. If the stimulation frequency tracks the resonance frequency^75^, at a certain frequency of stimulus, brain response jumps and large amplitude oscillations occur. As the amplitude of oscillation increases, the resonant frequency decreases up to a certain point in which the brain response returns to a small amplitude oscillation regime. So, based on this model, in an epileptic brain, internal resonance tracking loop brings the system to resonance, but nonlinearity leads to a hysteresis loop and so the brain dynamics jumps between small amplitude oscillations in interictal state and large amplitude oscillations in ictal state. These transitions are illustrated schematically in Fig. 8.

The proposed dynamical scenario of state transitions has some interesting features that are in agreement with EEG findings for common types of seizures such as tonic–clonic and absence seizures. Abrupt onset and abrupt termination of the seizures, progressive increase in amplitude and decrease in frequency during ictal phase^76–79^ and sensitivity to rhythmic stimulations such as flashing or light flickering at certain frequencies can be explained by this conceptual model. Epileptic seizure in photosensitive epilepsy patients is most frequently induced by 15 Hz flicker stimulus when the eyes are open^80^ and 10 Hz stimulus when the eyes are closed^81^, which can confirm the resonance nature of epileptic seizures. Based on this hypothesis, one can also expect to induce seizure in animal models more effectively by choosing the stimulation frequency close to the resonance frequency of their brains^82,83^. In addition, factors and conditions that change the brain state to a relatively lower frequency and higher amplitude oscillations (e.g. depression or sleep) can increase the probability of the nonlinear resonance in the brain. Such factors can be considered as epileptic seizures activators.

The main motivation of the present work was to take a step towards a dynamic description of epileptic seizures. The deterministic mechanism of seizure generation proposed in this paper, paves the way for further studies on possible seizure prevention approaches.
